# Histological and Lectinhistochemical Pattern of the Inflammatory Response to *Ascocotyle* sp. Metacercariae in *Mugil liza* Hearts

**DOI:** 10.3390/ani16182939

**Published:** 2026-09-18

**Authors:** Luca Di Cesare, Claudio G. Barbeito, Jorge Barneche, Martín M. Montes, Silvia E. Plaul

**Affiliations:** 1 Instituto de Morfología y Patología Animal (IMPA), Facultad de Ciencias Veterinarias, Universidad Nacional de La Plata, Buenos Aires 1900, Argentina; 2 Centro de Estudios Parasitológicos y Vectores (CEPAVE, CCT, CONICET), Consejo Nacional de Investigaciones Científicas y Técnicas, Universidad Nacional de La Plata, Buenos Aires 1900, Argentina; 3 Cátedra de Bioquímica, Facultad de Ciencias Veterinarias, Universidad Nacional de La Plata, Buenos Aires 1900, Argentina

**Keywords:** ventricle, atrium, PHA-L lectin, macrophages, mugilids

## Abstract

*Mugil liza*, an economic resource fish species along the South American Atlantic coast, serves as the sole mullet species breeding in Argentina. While encysted parasites in mullets typically target the bulbus arteriosus, this study identifies *Ascocotyle* sp. metacercariae localized exclusively within the cardiac ventricle of *Mugil liza*. Thirty juvenile specimens were collected from Samborombón Bay, Argentina, to evaluate the resulting cardiac lesions and associated inflammatory host response through histological, lectinhistochemical, and immunohistochemical analyses. Pathological evaluation revealed marked disruption of the myocardial architecture, characterized by regions of coagulative necrosis, presence of melanomacrophage centers (MMCs), and congested blood vessels containing cellular debris within their lumen. A persistent mononuclear inflammatory infiltrate—comprising eosinophilic granular cells, macrophages, and lymphocytes—was observed surrounding the ventricular cysts and extending into the atrium. Lectinhistochemical characterization identified a shared glycan profile, marked by positivity for *Phaseolus vulgaris* leucoagglutinin (PHA-L) and *Pisum sativum* agglutinin (PSA), across monocyte and macrophage lineages. This specific carbohydrate expression highlights the active participation and recruitment of these immune cell populations during the host defense. Overall, these pathological and glycobiological findings demonstrate that *Ascocotyle* sp. metacercariae act as a chronic immunogenic stimulus, driving and sustaining localized myocardial inflammation in *M*. *liza*.

## Simple Summary

Fish, like all animals, defend themselves against injury and infection by sending immune cells to the affected organ. Some parasites spend part of their life cycle inside fish, where they form cysts in various organs, including the heart. In the mullet’s heart, these cysts are usually reported in the bulbus arteriosus, and only rarely in its main muscular chamber, the ventricle. We found such cysts in the ventricle of the lebranche mullet, a species of commercial value in South America, and examined how the fish responded to them, using several staining methods, including one based on proteins that bind to specific sugar molecules on cells. We identified four types of immune cells around the cysts and successfully distinguished two of them for the first time in this species. We also found that prolonged inflammation damaged the surrounding heart muscle, an effect not previously described in this fish. These results clarify how mullets react to parasites and provide a reference point for assessing the health of wild and farmed populations.

## 1. Introduction

Members of the family Mugilidae constitute one of the main resources in commercial estuarine fisheries throughout tropical, subtropical and temperate seas [1]. *Mugil liza* Valenciennes is distributed along the Atlantic coast of South America, from Argentina to Venezuela [2]. This species is an important economic resource [3]. Furthermore, it is the only mullet species that breeds in Argentina [4].

Heterophyiasis is a zoonosis transmitted to humans through the consumption of raw, undercooked, or inadequately smoked fish containing parasite metacercariae. This disease occurs in many parts of the world, and is endemic to Southeast Asia [5,6]. One of the most significant genera associated with this disease is *Ascocotyle* Loss, whose metacercariae have been found encysted in various organs (gills, bulbous arteriosus, mesentery, skeletal muscles, liver and intestine) of marine, brackish, and freshwater fish [7,8,9]. In Argentina, the only *Ascocotyle* species identified in *M*. *liza*, based on morphology and molecular biology, was *Ascocotyle (Phagicola) longa* Ransom, 1920 [10,11]. No studies have been reported on the histopathological lesions caused by *Ascocotyle* sp. metacercariae in the atrium or ventricle of *M. liza*; however, Nolan et al. [12] and Warren et al. [13] reported other digenean species parasitizing the ventricles of teleosts.

Parasites can induce various inflammatory responses in fish organs [14]. Among the main cellular components involved in these responses are eosinophilic granular cells (EGCs) [15,16,17] and melanomacrophage centers (MMCs). The latter are groups of macrophages with pigments such as lipofuscin, melanin and hemosiderin, which, under normal conditions, can be found in various organs such as cranial kidney, spleen and liver of teleost fish [18,19]. However, an increase in their size or number, or their presence in non-habitual organs, indicates a distress condition. Studies have analyzed the pattern of glycoconjugates involved in the fish immune response using lectinhistochemistry (LHC) [20,21]. In these studies, phagocytic cells and leukocytes exhibit high affinity for mannose, glucose, and galactose residues [18]. Lectins may vary depending on the cell type involved; thus, this technique is used to locate, identify, and differentiate variations in the saccharide residues present in glycoconjugates to discriminate different cell populations [22]. Certain lectins, such as *Phaseolus vulgaris*-L (PHA-L), have been employed as markers for immune system cells [23,24].

To date, the inflammatory response against *Ascocotyle* sp. in the heart of mugilid fish remains unclear. The objectives of this work are to describe the heart lesions in parasitized specimens of *M. liza* through the evaluation of histological sections, and to characterize the inflammatory response associated with metacercariae of *Ascocotyle* sp.

## 2. Material and Methods

### 2.1. Animals

The specimens used in this work were collected according to the Ethics Committee for the Use of Animals (CICUAL) of FCV, UNLP, animal welfare laws, guidelines and policies as approved by the CD and FCV under the protocol code 111-1-20T and date of approval 22 March 2022.

Thirty juvenile specimens of *M*. *liza* (total length 15.38 ± 2.67 cm; standard length 13.97 ± 2.74 cm; body weight 31.98 ± 0.84 g) were collected during a parasitological survey carried out in Punta Rasa, Samborombón Bay (36°17′23″ S and 56°46′53″ W) (Buenos Aires, Argentina). Fish were collected using a modified Garlito/Bituron stationary trap net [25] and a seine net (10 m in length with a stretched mesh size of 5 mm at the wings and 2.5 mm at the codend). Animals were euthanized using an anesthetic overdose of Eugenol (30 mg L^−1^) [26]. Necropsies were performed immediately after euthanasia; hearts were removed and examined under a Luxeo 6Z binocular stereomicroscope (Labomed 4146100−800, Fremont, CA, USA). To identify the metacercariae species, some of them were extracted, examined and compared with the description by Martorelli et al. [10]. Non-parasitized hearts were used as negative controls.

### 2.2. Histological Processing

Samples were fixed in 10% buffered formalin and using standard paraffin embedding histology procedures, following our laboratory protocol [27]. Slices 5 μm thick were obtained with a sliding microtome (Optische Werke C. Reichert, Viena, Austria, No. 17774) and stained with hematoxylin-eosin (H&E) for routine examination, and Masson’s Trichrome to differentiate collagen fibers from muscle fibers [28].

### 2.3. Lectinhistochemistry

The mounted sections were deparaffinized, rehydrated, and then incubated with 0.03% H_2_O_2_ in methanol (purum P99.0%) for 30 min at room temperature to inhibit endogenous peroxidase activity. The slides were then treated with 1% bovine serum albumin (BSA) in 0.2 M PBS for 30 min and incubated at 4 °C overnight with seven different specific biotinylated lectins Vector Laboratories, Inc. (Burlingame, CA, USA) (Table 1). The concentration of the lectins utilized was 15 µg/mL. Diaminobenzidine (DAB, DAKO K346711-2, Agilent Technologies, Inc., Santa Clara, CA, USA) was used as chromogen and tap water was used to cut off the reaction and then switched to distilled water. Hematoxylin was used as a nuclear counterstain. Staining intensity was graded according to a semiquantitative range (−) negative reaction, (1) weakly positive, (2) moderately positive, and (3) strongly positive. Lectin staining controls included exposing samples to substrates without lectin and incubation samples with lectins pre-incubated with their corresponding sugar inhibitors [23].

### 2.4. Immunohistochemistry

To locate proliferating cells, selected histological sections were processed by immunohistochemical techniques using antibodies against proliferating cell nuclear antigen (PCNA). The slides were dried and primary monoclonal anti-PCNA antibody (dilution 1/3000, Cat #SAB2701819, RRID: AB_2819173; Sigma-Aldrich, Ciudad Autónoma de Buenos Aires (CABA), Argentina). Next, the slides were subjected to three washes in PBS for 5 min each, then drained and dried. Dako EnVision+ System—HRP Labeled Polymer Anti-mouse (cod k4001) was used as secondary antibody and amplification of reaction system incubated in a humidity chamber for 15 min. This was followed by two 5 min PBS washes. The samples were followed by two 5 min PBS washes. Negative control sections were prepared by omitting the primary antibody, whereas positive control sections were samples of mammalian intestine. Diaminobenzidine (DAB, DAKO K346711-2) was used as chromogen and tap water was used to cut off the reaction and then switched to distilled water. Hematoxylin was used as a nuclear counterstain [28].

All histological sections were observed with a Leica DM1000 microscope (Wetzlar, Germany), and digital photomicrographs and measurements were acquired using an image capture system (Leica ICC50W, Wetzlar, Germany).

## 3. Results

Trematode metacercariae were found in sixteen mullets and only in the heart ventricle; these were identified as *Ascocotyle* sp. according to the morphological characteristics of the number of hook rows and number of hooks [10].

**Histological characterization:** Non-parasitized mullet hearts (N: 14) exhibited normal microscopic structure. The endocardium of the sinus venosus and atrium consisted of endothelium and striated cardiac muscle.

**Figure 1:**
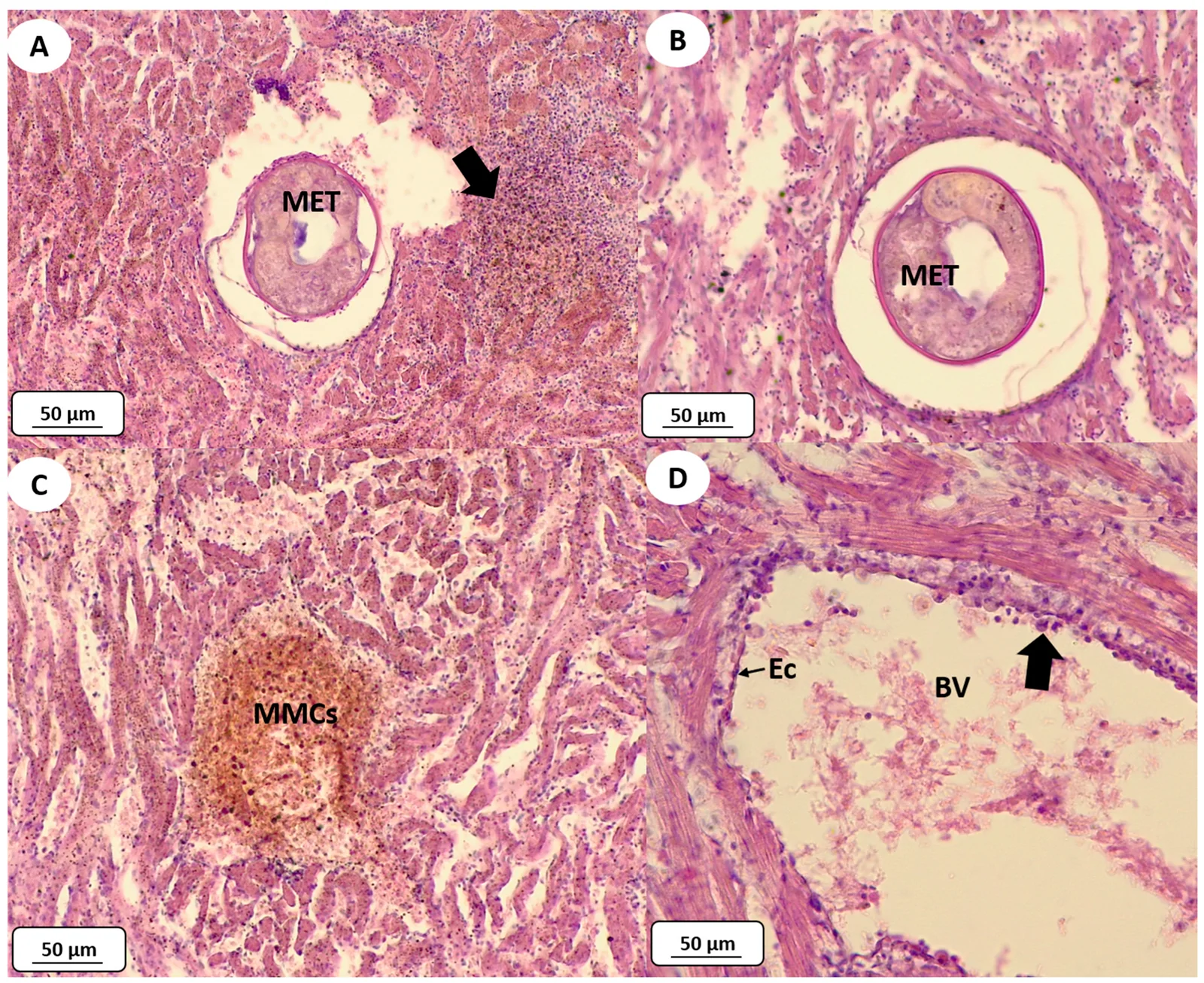
**Ventricular myocardial metacercariae location and associated inflammatory response, H&E.** (**A**,**B**) Encysted metacercariae and mononuclear cell infiltrate (black arrow) composed of lymphocytes and macrophages. (**C**) Areas showing melanomacrophage centers (MMCs), loss of tissue architecture, and pigment aggregation among cardiomyocytes. (**D**) Detailed view of a dilated blood vessel with EGCs, lymphocytes, and monocytes in close contact with the endothelium. Ec: endothelial cells; MET: metacercariae; BV: blood vessel; black arrow: mononuclear cell infiltrate.

**Figure 2:**
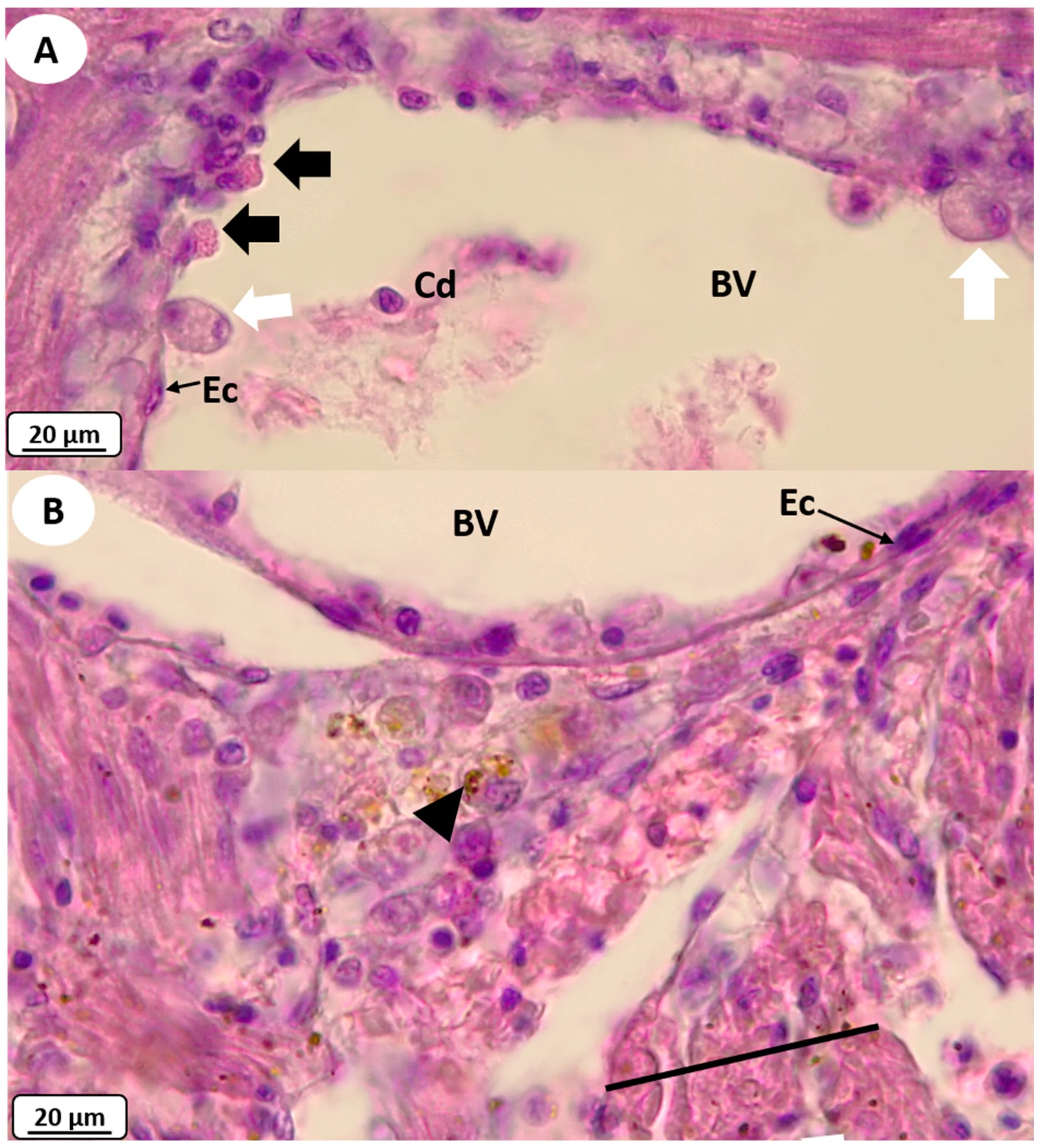
**Inflammatory response in the myocardium and intraventricular blood vessels, H&E.** (**A**) Multiple EGCs and monocytes in contact with the endothelium, as well as cellular debris (Cd) within the vascular lumen. (**B**) A group of pigmented macrophages (arrowhead) in close contact with blood vessels. The black line indicates an area of necrosis with fibrillar disorganization of cardiomyocytes. Ec: endothelial cells; BV: blood vessel; black arrow: EGCs; white arrow: monocytes.

**Figure 3:**
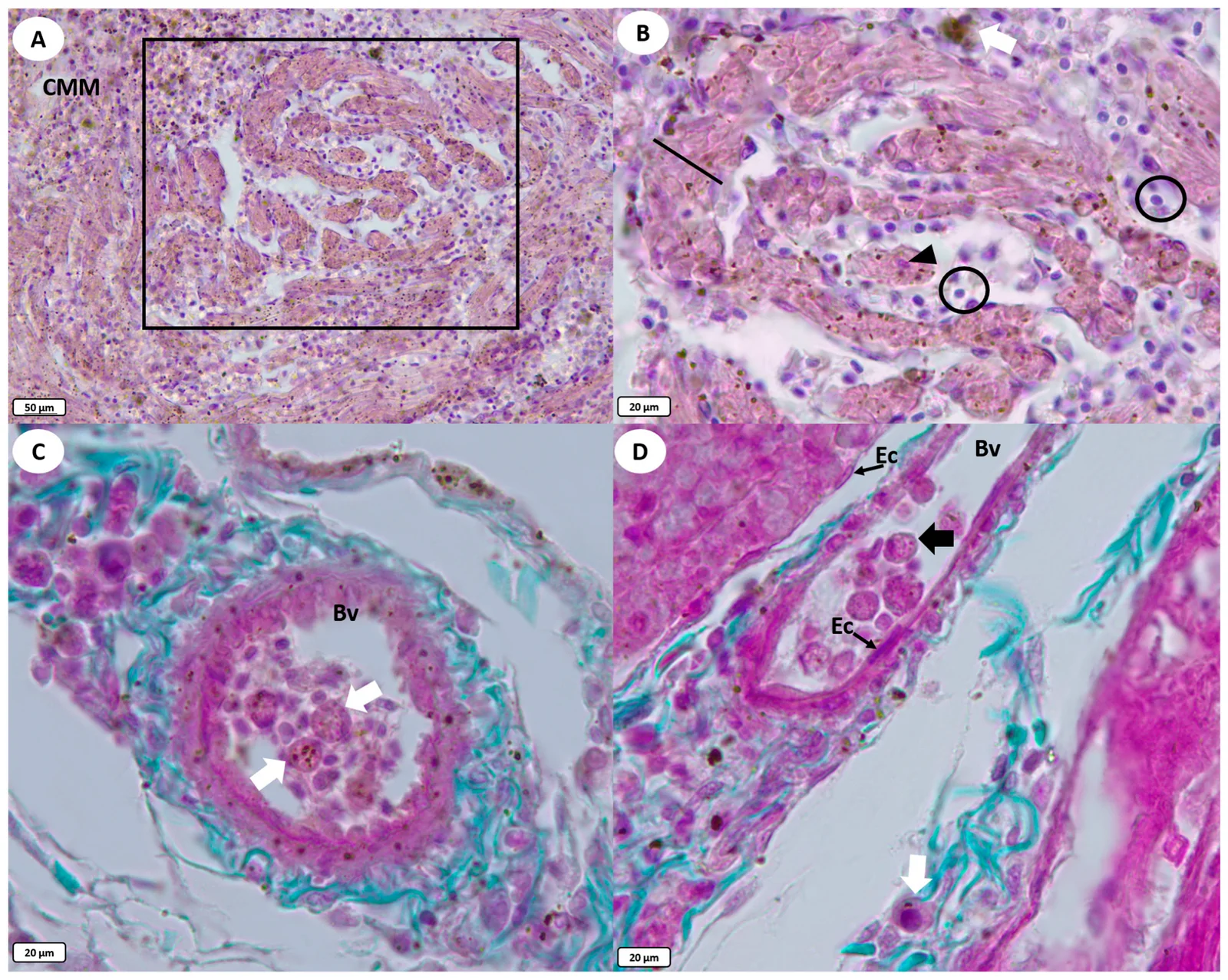
**Histopathological findings in the ventricular myocardium and epicardial blood vessels.** (**A**) Ventricular myocardium showing an area of coagulative necrosis (black box), myofibrillar disorganization, lymphocytic infiltrate, and MMC formation adjacent to the injury. H&E. (**B**) Detail of the necrotic area, cardiomyocytes (black line) show loss of cellular architecture, pyknotic nuclei (arrowhead), and infiltrating lymphocytes (circles) among cardiomyocytes. H&E. (**C**,**D**) Monocytes (white arrows) with cytoplasmic brown pigments. Masson’s Trichrome. Bv: blood vessel; Ec: endothelial cell; black arrow: EGC; Melanomacrophage center: CMM; black circle: lymphocyte.

**Figure 4:**
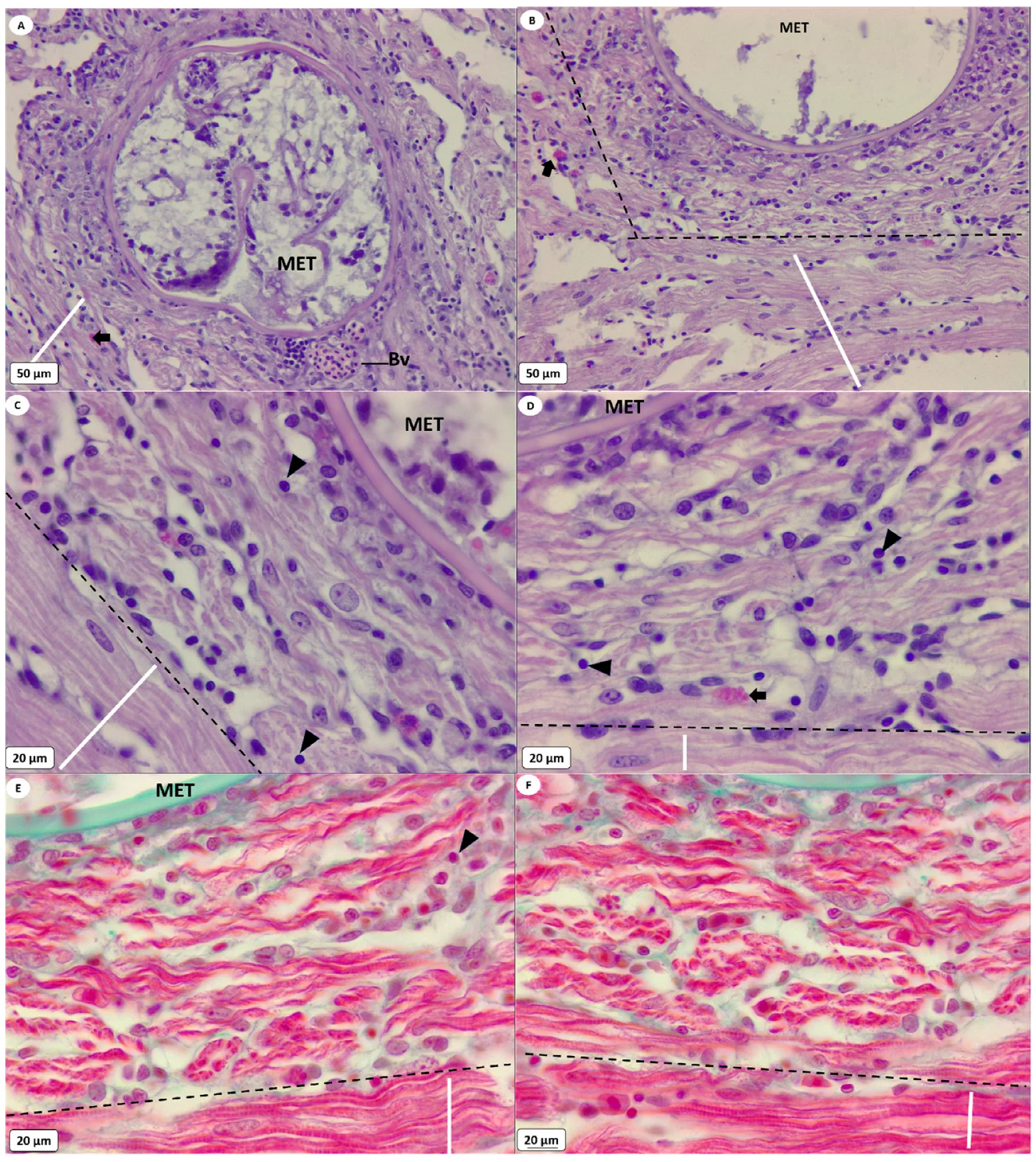
**Atrophic morphological changes in the myocardium associated with the inflammatory response.** (**A**) Encysted metacercaria in the myocardium; note a congested blood vessel. H&E. (**B**) Areas of myofibrillar disorganization associated with metacercaria. H&E. (**C**,**D**) Disorganization of cardiomyocytes with loss of normal cross-striations and lymphocytic infiltration among myofibers. H&E. (**E**,**F**) Detail showing granular sarcoplasm in cardiomyocytes and increased collagen deposition. Masson’s Trichrome. Arrowhead: lymphocytes, black arrow: granular eosinophilic cell; MET: metacercariae; (Bv/black line): blood vessel; black dashed line: myocardium with atrophic changes, white line: myocardium without lesions.

In parasitized mullets, the metacercariae invaded the cardiac muscle and were encapsulated by a thin layer of connective tissue (Figure 1A,B). Melanin deposits were observed within cardiomyocytes, alongside cardiac muscle disorganization surrounding the cysts and a mononuclear inflammatory infiltrate composed of EGCs, macrophages, and lymphocytes (Figure 1A). Large MMCs were also observed (Figure 1C), as well as pigment deposits between cardiomyocytes (Figure 1 and Figure 2). In certain regions of ventricular myocardium, circumscribed areas of coagulative necrosis were observed (Figure 3A,B). Within the necrotic areas, cardiomyocytes were characterized by the absence of nuclei and alterations in their morphological and staining properties, such as loss of typical striations due to myofibrillar disorganization, granular-appearing sarcoplasm, and even in some cells, pyknosis and karyolysis (Figure 3B). In the ventricular epicardium, thickened and congestive blood vessels were observed; additionally, a mononuclear cell infiltrate composed of lymphocytes and macrophages was identified (Figure 3C,D). Furthermore, myocardial fibers in areas adjacent to the inflammatory response showed atrophic changes (Figure 4A–D). The cytoplasm of some cardiomyocytes lacked typical striation compared to more distant regions (Figure 4C,D), displaying a granular appearance and discontinuities (Figure 4E,F). An increase in connective tissue collagen fibers was observed between the cardiac muscle bundles (Figure 4E,F).

**Figure 5:**
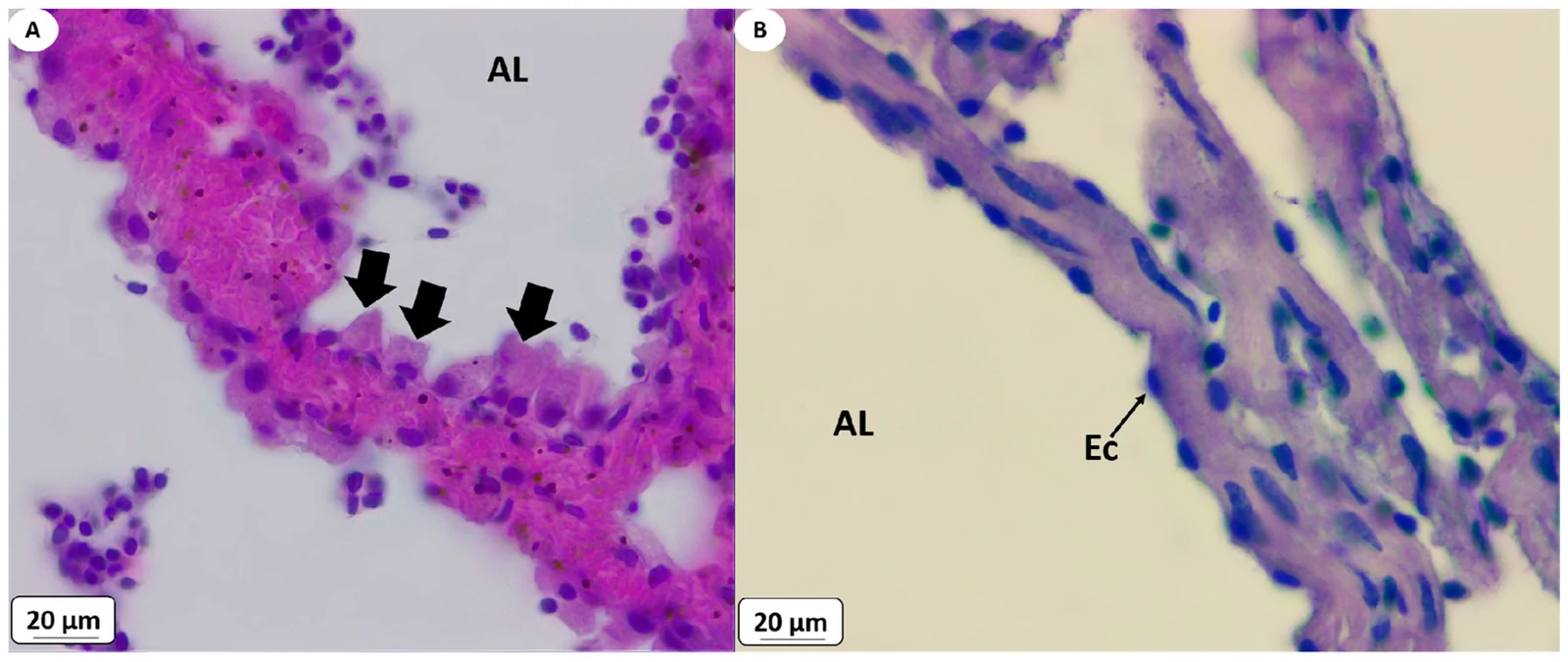
**Morphological characterization of monocytes in contact with the atrial wall.** (**A**) Monocytes attached to the atrial endothelium. H&E. (**B**) Atrial endothelium of a non-parasitized heart. H&E. AL: atrial lumen; Ec: atrial endothelium; thick black arrows: monocytes.

In six hearts, in addition to the ventricular inflammatory infiltrate, monocytes with sponge-like cytoplasm in contact with the atrial endothelium were identified (Figure 5A). These cells formed aggregates, which were in palisade-like arrangement opposite to endothelial cells of the endocardium.

**Figure 6:**
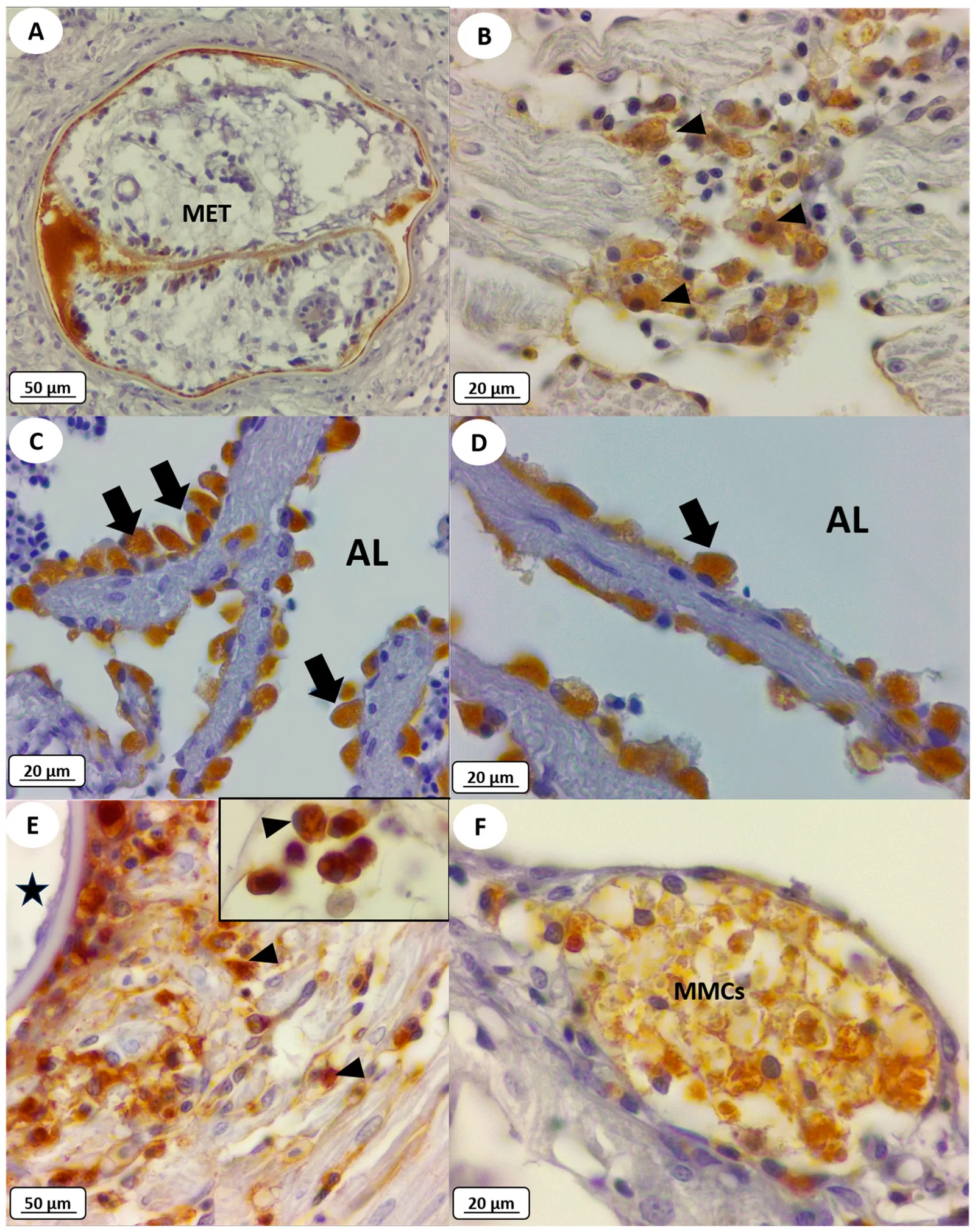
**Lectinhistochemical characterization of the inflammatory response in the atrium and ventricle.** (**A**) Metacercarial capsule showing a moderate positive reaction to PNA. (**B**) Macrophage infiltrate scattered throughout the ventricular myocardium, showing a moderate positive reaction to PSA. (**C**,**D**) Monocytes arranged in a palisade pattern on the atrial endothelium, showing a strong positive reaction to PHA-L. (**E**) Encysted metacercaria in the ventricular myocardium, with scattered macrophages showing a strong cytoplasmic positive reaction to PHA-L; the inset shows a higher-magnification view. (**F**) MMC in the ventricular myocardium, showing a moderate positive reaction to PHA-L. Black arrowhead: dispersed macrophages; MMCs: melanomacrophage centers; AL: atrial lumen; black arrows: monocytes; star/MET: metacercariae.

**Lectin histochemical characterization**: The application of lectin histochemistry to detect glycoconjugates involved in the immune response of fish revealed a similar pattern in both regions studied. In metacercariae, as well as in the cytoplasm of monocytes/macrophages and MMCs, no labeling was observed in either of these regions with SBA, WGA, LCA, and RCA-I lectins. The results are summarized in Table 2. Metacercariae: PNA lectin was the only one that exhibited a specific and moderate labeling pattern in both the metacercariae and their capsule (Figure 6A), demonstrating the presence of terminal galactose residues.

Monocytes/macrophages: PHA-L lectin produced an intense and highly specific labeling in atrial monocytes and ventricular macrophages (Figure 6C–E), suggesting a high abundance of branched N-glycans in the glycoproteins of these cells. In ventricular macrophages, moderate cytoplasmic labeling was also observed with SJA and PSA lectins (Figure 6B), indicating the presence of N-acetylgalactosamine and D-mannosyl residues; in contrast, atrial monocytes showed reduced labeling for PSA lectin and were negative for SJA lectin (Supplementary Materials Figures S1 and S2).

MMCs: PHA-L, SJA, and PSA lectins showed the same positive labeling pattern observed in ventricular macrophages; however, the labeling intensity for all three lectins was moderate within MMCs (Figure 6F). Consequently, a slight decrease in the abundance of branched N-glycans in glycoproteins of melanomacrophage cells forming MMCs was observed compared to ventricular macrophages (Supplementary Materials Figures S1 and S2).

**Proliferating Cell Nuclear Antigen (PCNA)**: In both parasitized and non-parasitized hearts, cardiomyocytes nuclei in the atrium and ventricular myocardium were negative for anti-PCNA antibody.

## 4. Discussion

There are few studies on *Ascocotyle* metacercariae encysted in the ventricle of mugilids [29]. Although Martorelli et al. [10] identified *Ascocotyle (Paghicola) longa* metacercariae in the heart of *M*. *liza*, their study did not specify the exact cardiac region where encysted metacercariae occurred, nor did it describe the histopathological lesions. Our study is the first report on the type of inflammatory response against *Ascocotyle* sp. metacercariae in the atrium and ventricle of *M*. *liza*. To date, the only previously reported lesions were edema and hemosiderin deposition among ventricular cardiomyocytes [29,30]. However, our findings reveal blood vessel dilation and a mononuclear inflammatory cell infiltrate composed mainly of EGCs, lymphocytes, and macrophages. This involvement of macrophages in different immune responses is consistent with Mokhtar et al. [18]. The recruitment of EGCs against different parasitic agents is a common cellular response in teleosts [17,18,31,32]. This is consistent with our results regarding the considerable number of EGCs both inside and outside distended ventricular blood vessels; this vascular distention could be related to vasodilatation caused by EGC degranulation [14]. In the present study, all metacercariae were encysted in the ventricle, and no abnormalities were observed in the bulbus arteriosus. Since the lumen of the arterial bulb remained unobstructed, hemodynamic changes were probably absent. Nevertheless, further studies assessing parameters such as heart rate and partial pressure of oxygen in parasitized individuals would help to confirm this.

Granulomatous reactions against metacercariae in fish have been described with varying degrees of complexity. In *Sphyraena viridensis* Cuvier, up to five distinct granuloma stages were characterized during parasitic infection [33]. Conversely, Polinas et al. [34] proposed a three-stage classification: Stage I (pre-granuloma), Stage II (intermediate), and Stage III (advanced granuloma) focused on *Ascocotyle* lesions in the bulbus arteriosus of *Mugil cephalus* Linnaeus. However, these studies lack specific host morphometric data, such as total or standard length, weight, or age, which hinders direct comparisons with host developmental status. We have found that a granulomatous lesion in different stages characteristic of chronic inflammations like that reported by Polinas et al. [34] coexisted within the ventricular myocardium of the same individual. This spatio-temporal overlap of early and advanced inflammatory responses strongly suggests that *M*. *liza* experiences multiple, non-simultaneous infection events throughout its life cycle. Another key difference observed was the lesion type in the ventricular myocardium. In contrast to those reported by Polinas et al. [34], our study identified focal areas exhibiting atrophic changes and necrosis. However, Steinbach et al. [35] did report this potential type of myocardial injury. Although the atrophic changes observed in the *M. liza* myocardium do not correspond to those described by Polinas et al. [34], this discrepancy could be attributed to several factors. The first is a more pronounced cellular inflammatory response against metacercariae. Indeed, our findings revealed a marked mononuclear inflammatory infiltrate, characterized by the involvement of lymphocytes and macrophages in response to the parasite. These disparities could also stem from interspecific differences among the studied mullet species.

Very few studies report myocardial damage in hearts parasitized by the genus *Ascocotyle*. Research conducted by Hicks and Steele [30] on the hearts of another euryhaline fish, *Fundulus heteroclitus* Linnaeus, parasitized by *Ascocotyle tenuicollis* Price, showed no alterations in myocardial tissue organization such as those observed in *M. liza*.

Several macrophage phenotypes have been described in teleosts, among which the M1 activation state is the best characterized. These cells play a fundamental role in host defense by rapidly eliminating pathogens through phagocytosis, phagolysosomal acidification, nutrient restriction, and the generation of toxic reactive species [32]. These cells can rapidly eliminate pathogens through phagocytosis and the production of toxic reactive intermediates, phagolysosomal acidification, and the restriction of nutrient availability [32]. In helminthiasis or tissue parasitosis, such as encysted metacercariae, M1 macrophages dominate the acute or initial phase of granuloma formation, surrounding the parasite to destroy or contain it [36].

Different cytokines produced during the inflammatory response promote the recruitment and differentiation of macrophages [37,38]. A notable finding observed in our study was the accumulation of monocytes on the atrial endothelium. This specific location may represent a key step in host pathogen recognition and subsequent extravasation toward the inflammatory site within the ventricular myocardium. In addition to the involvement of macrophages as solitary cells in the inflammatory process, MMCs were also found in the ventricular myocardium. The presence of several MMCs associated with metacercariae, as well as in other regions of the ventricular myocardium, represents a distinction between our findings and those reported by other authors [29,30,34].

However, we found no reports regarding the presence of MMCs of varying sizes and containing a range of pigments within the ventricular myocardium. MMCs contain melanin and related pigments, which are thought to play a defensive role in many organisms due to their capacity to generate hydrogen peroxide [39]. MMCs are also a potent bactericidal structure and may possess additional immunological properties, such as facilitating wound healing [40]. The presence of pigment deposits in the ventricle of the mullets in our study is likely associated with the nature of the inflammatory response to metacercariae.

During infection, resident macrophages detect tissue damage or infiltrating pathogens, such as parasites, through extracellular or intracellular pattern recognition receptors [41]. Some parasites, such as those of the genus *Ascocotyle*, possess an encysted metacercaria that protects them from the external environment [9,10]. The composition of this cyst may include glycoconjugates capable of being recognized by the host’s immune system [15]. Lectins are proteins that recognize specific carbohydrate or glycan residues. These glycans are associated with lipids and proteins, both intracellularly in the cytosol or within certain organelles and extracellularly, allowing for the identification of specific glycoconjugates [23]. In our study, high affinity for PNA lectin was observed in the metacercarial cyst of *Ascocotyle*, demonstrating the presence of β-galactose residues. The glycoconjugate pattern present in metacercarial cyst from *M. liza* could act as a pathogen-associated molecular pattern and promote the inflammatory response observed in the myocardium [37,38]. Additional studies using additional variety of lectins could provide new information regarding the composition and possible modification of the glycoconjugates present in the parasite.

PHA-L binds to key cell-surface glycoproteins in monocytes/macrophages, such as integrins (CD11b/CD18), CD44, or cytokine receptors, thereby affecting cell adhesion and migration. Contrary to the findings reported by Castagnaro et al. [24], our results revealed strong positive labeling for PHA-L in monocytes/macrophages located in both the atrium and ventricle, respectively. Imagawa et al. [20] analyzed various immune system cells using lectin histochemistry to evaluate their glycan profiles. Consistent with our findings, their results demonstrated that macrophages were positively labeled with PHA-L and PSA lectins, confirming the presence of complex N-glycans and α-mannose residues. According to Bojar et al. [23], positivity for PSA lectin may be associated with cellular cytotoxicity and inflammation. In their study, Castagnaro et al. [24] observed that PNA and RCA-I lectins showed strong positive staining in the cytoplasm of macrophages present in rainbow trout kidneys. However, in both our study and that of Imagawa et al. [20], PNA lectin was completely negative. The observed differences in macrophage glycoconjugate expression are likely attributable primarily to interspecies variations among fish, the specific organ analyzed, or the nature of the inflammatory stimulus and its corresponding specific immune response.

Knowledge regarding cell proliferation in the fish myocardium is currently limited. PCNA, a highly conserved eukaryotic protein, shows robust expression indicative of DNA replication or repair, a phenomenon broadly described in fish under normal and pathological states [42]. In contrast to Yousaf et al. [43], who observed increased PCNA positivity in diseased hearts, cardiomyocyte nuclei in our study were completely PCNA-negative. This finding is consistent with the absence of extensive myocardial necrosis in our histopathological analysis, suggesting that the level of damage fell below the threshold required to activate cardiomyocyte cell cycle re-entry. Further research will be essential to clarify whether non-proliferative signaling pathways in cardiomyocytes are involved in the tissue response to injury.

The inflammatory response to tissue injury is essential for tissue repair, including in the heart. In fish such as zebrafish, this response not only regenerates lost cardiomyocytes but also remodels and resolves cardiac scarring [44]. Macrophages play a pivotal role in scar formation and resolution following cardiac injury, as demonstrated in both zebrafish and adult mice [37,38]. In the present study, the inflammatory response in the analyzed hearts was characterized by a marked presence of both dispersed macrophages and those organized into MMCs. The activity of these macrophages is likely also linked to healing and tissue repair at the injury site. Indeed, according to Noga et al. [45], the persistence of antigens that are highly resistant to cellular digestion can drive the differentiation of macrophages into distinct functional phenotypes.

These results are consistent with our findings regarding the presence of macrophages in the immune response. Lectin histochemistry analysis of the glycoconjugate pattern demonstrated that atrial wall-adherent monocytes, dispersed macrophages, and MMCs expressed oligosaccharide residues recognized by both PHA-L and PSA, exhibiting distinct cytoplasmic labelling. Together, these data suggest that the metacercariae of *Ascocotyle* sp. may act as an agent that promotes and sustains a chronic inflammatory response in the ventricular myocardium of *M. liza*.

With respect to glycan expression profiles, dispersed myocardial macrophages, MMCs, and monocytes in contact with the atrial endothelium all expressed PHA-L reactive residues. Overall, lectinhistochemistry enabled the characterization of a shared PHA-L (+) glycans profile across the different monocyte and macrophage populations. Nevertheless, further studies are required to fully elucidate the variations in glycan expression among the diverse cell types involved in the host response to *Ascocotyle*. The presence of numerous MMCs in the ventricular myocardium of *M*. *liza*, accompanied by atrophic changes and necrosis, points to a distinct histopathological pattern induced by *Ascocotyle* sp. metacercariae.

## 5. Conclusions

These recently obtained data suggest that the metacercaria of *Ascocotyle* sp. may act as an agent that promotes and perpetuates a chronic inflammatory response in the ventricular myocardium of *M. liza*. The inflammatory infiltrate in the ventricle of *M. liza* was mainly composed of lymphocytes, eosinophilic granule cells, monocytes, and macrophages. Lectin histochemistry proved to be a useful tool for characterizing monocytes and macrophages, which showed positive cytoplasmic binding to PHA-L, the most reliable of the lectins tested. The presence of several MMCs in the ventricular myocardium of *M. liza*, combined with atrophic changes and necrosis, indicates a specific inflammatory response pattern to *Ascocotyle* sp. metacercariae.

## Figures and Tables

**Table 1:** Carbohydrate binding specificity of the lectins used in this study.

Lectins (Acronym)	Binding Specificity
*Phaseolus vulgaris* leucoagglutinin (PHA-L)	β1,6-branched N-glycans
*Glycine maximus* agglutinin (SBA)	N-acetyl-D-galactosamine
*Triticum vulgaris* agglutinin (WGA)	β(1 → 4)-N-acetyl-D-glucosamine
*Sophora japonica* agglutinin (SJA)	terminal α-β-linked N-acetylgalactosamine
*Arachis hypogaea* agglutinin (PNA)	terminal β-galactose.
*Pisum sativum* agglutinin (PSA)	terminal α-D-mannosyl residues
*Lens culinaris* agglutinin (LCA)	α-linked mannose residues
*Ricinus communis* agglutinin I (RCA-I)	β-D galactose

**Table 2:** Lectin-binding patterns in the inflammatory response and metacercariae.

Lectins	Macrophage/Monocyte	MMCs	MET
PHA-L	+++/+++	++	−
SBA	−/−	−	−
WGA	−/−	−	−
SJA	++/−	++	−
PNA	−/−	−	++
PSA	++/+	++	−
LCA	−/−	−	−
RCA-I	−/−	−	−

Staining intensity: (+++; strongly positive), (++; moderately positive), (+; weakly positive), (−; negative reaction). Melanomacrophage centers (MMCs), metacercariae (MET).

## Data Availability

The original contributions presented in this study are included in the article/Supplementary Materials. Further inquiries can be directed to the corresponding author.

## Supplementary Materials

The following supporting information can be downloaded at: https://www.mdpi.com/article/10.3390/ani16182939/s1, Figure S1: Lectin-histochemistry characterization of the inflammatory response; Figure S2: Lectin-histochemistry characterization of the inflammatory response and metacercariae.
