# Supplementary material for: Histological and Lectinhistochemical Pattern of the Inflammatory Response to Ascocotyle sp. Metacercariae in Mugil liza Hearts
Source: Animals (Basel). 2026 Sep 18;16(18):2939. doi: 10.3390/ani16182939 (PMC13603884; doi:10.3390/ani16182939)
Supplement: Supplementary file 1 [file animals-16-02939-s001.zip › animals-4440390-supplementary.pdf]

Supplementary Material

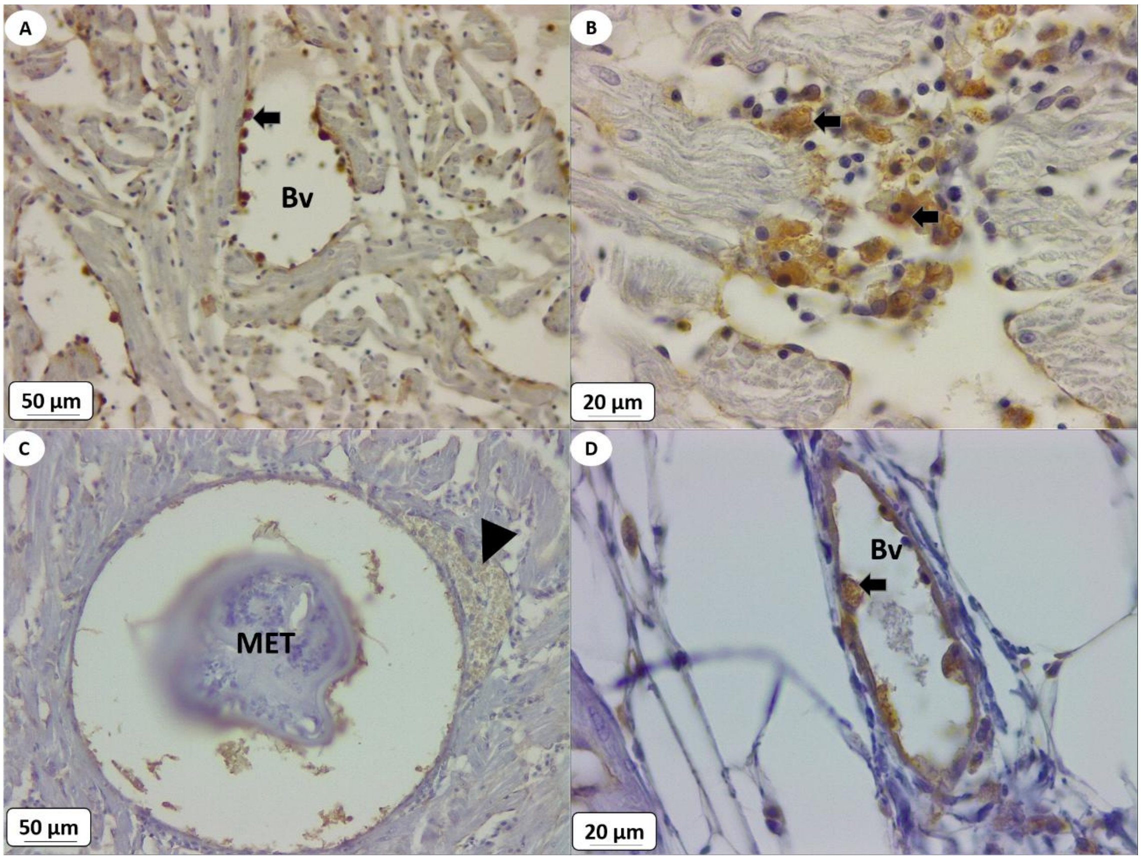

Figure S1: Lectin-histochemestry characterization of the inflammatory response.

A) Detail showing PSA (+) staining in monocytes in contact with a blood vessel wall. B) PSA (+) staining in the cytoplasm of macrophages in the myocardium. C-D) SJA (+) staining in MMCs and monocytes.

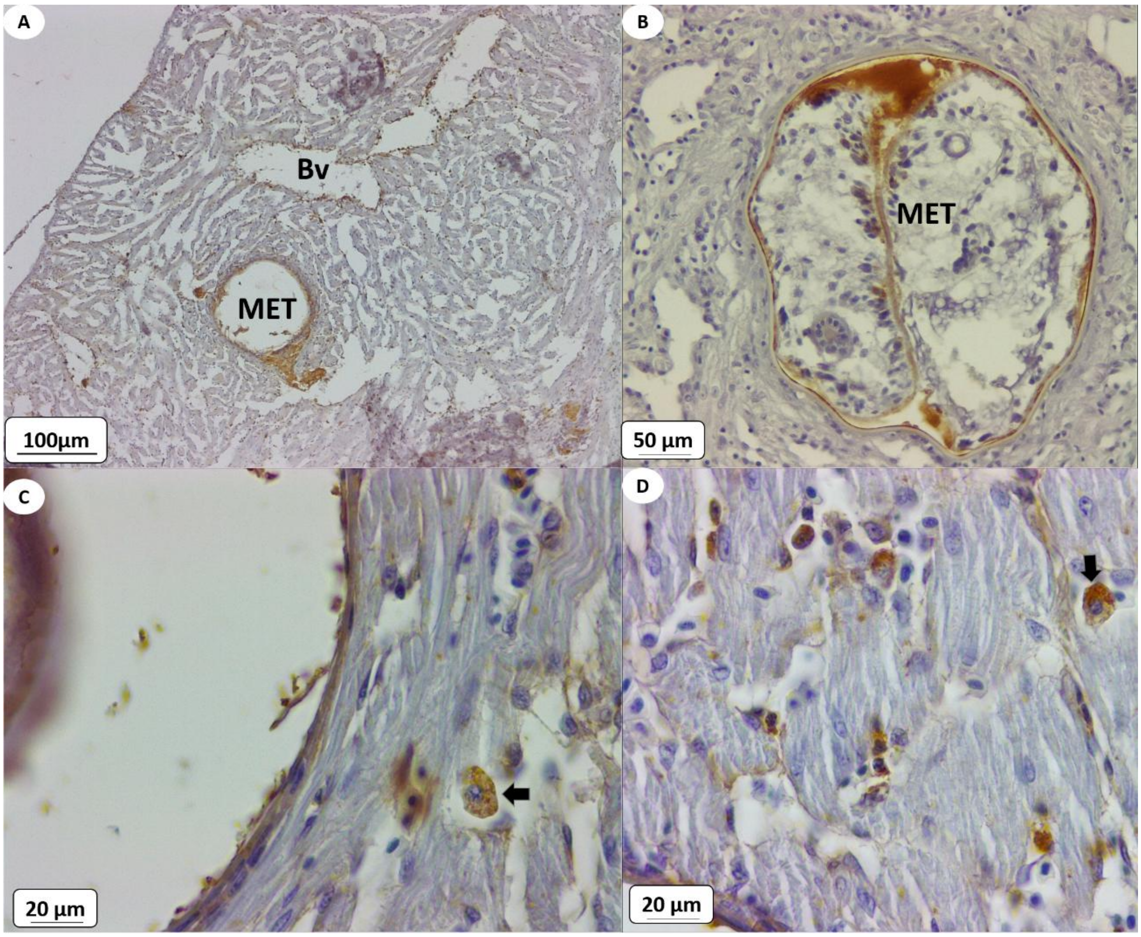

Figure S2: Lectin-histochemestry characterization of the inflammatory response and metacercarie.

A) PSA (+) staining in MMCs surrounding a metacercaria. B) PNA (+) staining in a metacercaria cyst. C-D) PSA (+) cytoplasmic staining in macrophages in the myocardium.
